# Progestin-primed ovarian stimulation protocol with or without clomiphene citrate for poor ovarian responders: a retrospective cohort study

**DOI:** 10.1186/s12905-022-02126-w

**Published:** 2022-12-17

**Authors:** Ahui Liu, Jie Li, Haofei Shen, Lili Zhang, Qiuyuan Li, Xuehong Zhang

**Affiliations:** 1 The First Clinical Medical College of Lanzhou University, The First Hospital of Lanzhou University, Lanzhou, Gansu China; 2 Key Laboratory for Reproductive Medicine and Embryo of Gansu Province, No. 1, Donggangxi Rd, Chengguan District, Lanzhou City, 730000 Gansu Province China; 3 The China-Japan Union Hospital of Jilin University, 126 Xiantai Street, Changchun City, 130033 Jilin Province China

**Keywords:** Progestin-primed ovarian stimulation, Poor ovarian response, Clomiphene citrate, Ovarian stimulation

## Abstract

**Objective:**

To explore the efficacy of progestin-primed ovarian stimulation (PPOS) combined with clomiphene citrate (CC) versus PPOS protocol used alone on cycle characteristics and pregnancy outcomes for women with the poor ovarian response (POR).

**Methods:**

We performed a retrospective cohort study and a total of 578 POR patients who underwent IVF/ICSI cycles were collected and divided into Group A (HMG 300 IU/d + MPA 10 mg/d) and Group B (HMG 300 IU/d + MPA 10 mg/d + CC 50 mg/d). The primary outcome measure was the number of oocytes retrieved, other outcome measures were cycle characteristics and clinical pregnancy rate.

**Results:**

The baseline information between the two groups were not statistically significant (*P* > 0.05). Compared with Group A, Group B had a lower total dose of human menopausal gonadotrophin (HMG) (2998.63 ± 1051.09 vs. 3399.18 ± 820.75, *P* < 0.001) and the duration of stimulation (10.21 ± 3.56 vs. 11.27 ± 2.56, *P* < 0.001). Serum luteinizing hormone level was higher in Group B on human chorionic gonadotrophin injection day (*P* < 0.001). The number of oocyte for retrieval, maturation, and fertilization were significantly lower in Group B than that in Group A (*P* < 0.001). However, the oocyte retrieval rate, maturation rate, fertilization rate, and viable embryo rate showed no statistical difference in the two groups (*P* > 0.05). After adjusting for confounders, the clinical pregnancy rate (OR 1.286; 95% CI 0.671–2.470) and live birth rate (OR 1.390; 95% CI 0.478–3.990) were comparable between the two groups.

**Conclusions:**

PPOS protocol combined with CC reduces the total dose of HMG and the duration of stimulation, and can also achieve similar oocyte yields and clinical pregnancy rate compared with the PPOS protocol used alone in poor ovarian responders.

## Introduction

Poor ovarian response (POR) indicates that the diminished ovarian reserve or poor response to ovarian stimulation, remains a challenge for both clinicians and patients in IVF/ICSI, which occurs in 5.6–35.1% of ovarian induction cycles [1]. These patients often face the problems of premature luteinizing hormone (LH) surge, lower oocyte yields, and higher cycle cancellation rate which caused emotional, physical, and financial burdens for infertile couples [2]. Due to the lack of consensus on the definition of POR in some previous clinical trials, the Bologna criteria was proposed by ESHRE and has been widely accepted for the definition of POR since 2011 [3]. To further improve the clinical diagnostic accuracy and management of POR patients, the POSEIDON criteria were established in 2016. According to this criteria, poor responders were divided into 4 groups based on age, ovarian response markers [antral follicle count (AFC) and anti-Müllerian hormone (AMH)], and response to induction [4].

Many studies are being devoted to finding a suitable and effective treatment to improve oocyte yields and clinical outcomes in women with POR. However, regardless of the mild or conventional ovarian stimulation strategies chosen, or co-treatment with clomiphene citrate (CC) or letrozole were found to be non-significant on oocyte yields and pregnancy outcomes, therefore, the most appropriate management of POR patients is still controversial, which needs further studies [1, 5]. Later, progestin-primed ovarian stimulation (PPOS) protocol was revealed to be effective and safe for suppressing premature LH surge and also showed similar oocyte yields and pregnancy outcomes compared with conventional short protocol [6, 7], which has been applied in normally ovulating women, poor responders, and polycystic ovarian syndrome (PCOS) [6, 8–10].

CC as the first drug to be used for the development of multiple follicles in ovarian stimulation regimens [11], is a selective estrogen receptor modulator, interfering with the negative feedback of endogenous estrogen on the hypothalamus-pituitary axis, and resulting in higher circulating concentrations of gonadotropin-releasing hormone (GnRH) [12]. PPOS protocol combined with CC was previously used in women with normal ovulatory and PCOS and reported that the addition of CC was beneficial in reducing human menopausal gonadotrophin (HMG) consumption and the duration of stimulation, alleviating the profound pituitary suppression caused by progesterone (P) to some extent, whereas did not conclusively improve oocyte yields and pregnancy outcomes [13, 14]. Data on the efficacy of CC in the PPOS protocol for POR patients are limited. Therefore, we conducted a retrospective cohort study in POR women to investigate the effectiveness of CC supplementation in the PPOS protocol during ovarian induction.

## Methods

### Data sources and patient selection

Data from this retrospective study were collected in women who underwent in vitro fertilization/intracytoplasmic sperm injection (IVF/ICSI) during the period January 2019 to June 2021 in our reproductive medicine center of the First Hospital of Lanzhou University. The study was approved by the First Hospital of Lanzhou University review boards. The inclusion criteria were that POR patients met the criteria of POSEIDON group 3 (Age < 35 years, AFC < 5 and AMH < 1.2 ng/ml) and group 4 (Age ≥ 35 years, AFC < 5 and AMH < 1.2 ng/ml), and the starting dose of HMG was limited to 300 IU. The exclusion criteria were patients with a history of pelvic tuberculosis, chromosome abnormality, severe endometriosis (meet the revised American Fertility Society (r-AFS) classification [15] III-IV stage during laparoscopic surgery), intrauterine adhesion, and other endocrine and metabolic diseases (poorly controlled diabetes or thyroid dysfunction). All patient details regarding diagnosis and treatment were available from our computer system. Finally, eligible patients were divided into two groups with or without CC supplementation during ovulation induction: 304 women in Group A (HMG 300 IU + MPA 10 mg), and 274 women in Group B (HMG 300 IU + MPA 10 mg + CC 50 mg).

### Ovarian stimulation procedures

All patients were scheduled to evaluate baseline hormones and do a transvaginal ultrasound on the third day of the menstrual cycle to determine ovarian stimulation protocols. All the patients received HMG (Lizhu Pharmaceutical Trading Co., China) 300 IU daily and Medroxyprogesterone acetate (MPA) (Zhejiang Xianju Pharmaceutical Co., Ltd.) 10 mg/d, starting from the third day of menstrual bleeding. Daily administration of CC (Fertilan; Codal-Synto Ltd., France) 50 mg was added in Group B and started from cycle day 3 onward. Transvaginal ultrasound was first performed on cycle days 7–8 to monitor the follicular growth, and serum follicle-stimulating hormone (FSH), luteinizing hormone (LH), estradiol (E2), progestin (P) were assayed for the same day. These checks were scheduled every two days, and when one or more dominant follicles reached a diameter of > 18 mm, trigger medicine 0.1 mg triptorelin (Decapeptyl, Ferring GmbH, Germany) and 2000 or 4000 IU human chorionic gonadotrophin (HCG) (Lizhu Pharmaceutical Trading Co., China) were administered. Oocyte aspiration was performed by transvaginal ultrasound guidance 34–36 h later.

Oocytes were fertilized in vitro by IVF or ICSI, depending on semen parameters on the day of oocyte aspiration. Embryos were evaluated on day 3 by the number and size of blastomeres and the degree of embryonic fragmentation [16], grade I and grade II embryos were considered as high-quality embryos that were cryopreserved by vitrification for frozen-thawed cycles. The spare non-high-quality embryos were further cultured until the blastocyst-stage was reached. At this stage, on days 5 or 6, only blastocysts with good morphology were frozen.

### Frozen embryo transfer (FET) cycle preparation

The embryos transferred in this FET cycle all came from the same ovulation induction cycle. There were 109 FET cycles in Group A and 88 FET cycles in Group B. Hormone replacement treatment was used in the present study for endometrial preparation. Oral estradiol valerate tablets (Bayer Healthcare Co., Ltd.) 2 mg were given three times a day starting from cycle day 3 for 10–14 days. Once endometrial thickness reached > 8 mm, oral dydrogesterone tablets (Abbott Biologicals B.V.) 30 mg/d and vaginal progesterone soft capsule (Cyndea Pharma, S.L.) 600 mg/d were started. The embryos were transferred on day 3 (cleavage stage) and day 5/6 (blastocyst stage). If clinical pregnancy was achieved, the progesterone supplementation was continued until 10 weeks of gestation.

### Hormone measurement

All hormone levels were determined by the chemiluminescence technique (Abbott Biologicals B.V.). Lower limits of sensitivity for detection were defined as follows: FSH = 0.06 mIU/ml, LH = 0.09 mIU/ml, E2 = 10 pg/ml, and *P* = 0.1 ng/ml. The upper limit for serum E2 detection was 5000 pg/ml. If the E2 concentration was higher than the upper limit on the trigger day or the day after, the E2 level was recorded as 5000 pg/ml.

### Statistical analysis

The primary outcome measure was the number of oocytes retrieved. Other outcome measures were cycle characteristics and clinical pregnancy rate. Clinical pregnancy was defined as the detection of an intrauterine gestational sac with a positive heartbeat on transvaginal ultrasound after 30 days of embryo transfer (ET). The miscarriage rate was defined as the proportion of patients with spontaneous loss of pregnancy before viability during early- and mid-pregnancy. Biochemical pregnancy was defined as a positive HCG test without visualization of an intrauterine gestational sac. Ongoing pregnancy was defined as a pregnancy beyond 12 weeks gestation. The gestational week was calculated according to the date of embryos were transferred.

Data were analyzed using the Statistical Package for the Social Sciences (SPSS, version 23.0) for Windows. Descriptive statistics of continuous variables of normal distribution were presented as mean ± standard and Student t test was used to compare two independent groups. The Mann–Whitney U-test for continuous variables of non-normal distribution. Categorical variables were expressed as numbers with percentages and compared using the χ^2^ test or Fishers exact test. *P* < 0.05 was considered statistically significant. Logistic regression analysis was performed to estimate the odds ratio (OR) and 95% confidence interval (CI) of pregnancy outcomes between the two groups. Accordingly, logistic regression analysis was also used to adjust the confounders, including age, AMH, BMI, AFC, E2 level on the day of HCG, retrieved oocytes, high-quality embryos, number of transferred embryos, and endometrial thickness.

## Results

### Patient characteristics

**Fig. 1:**
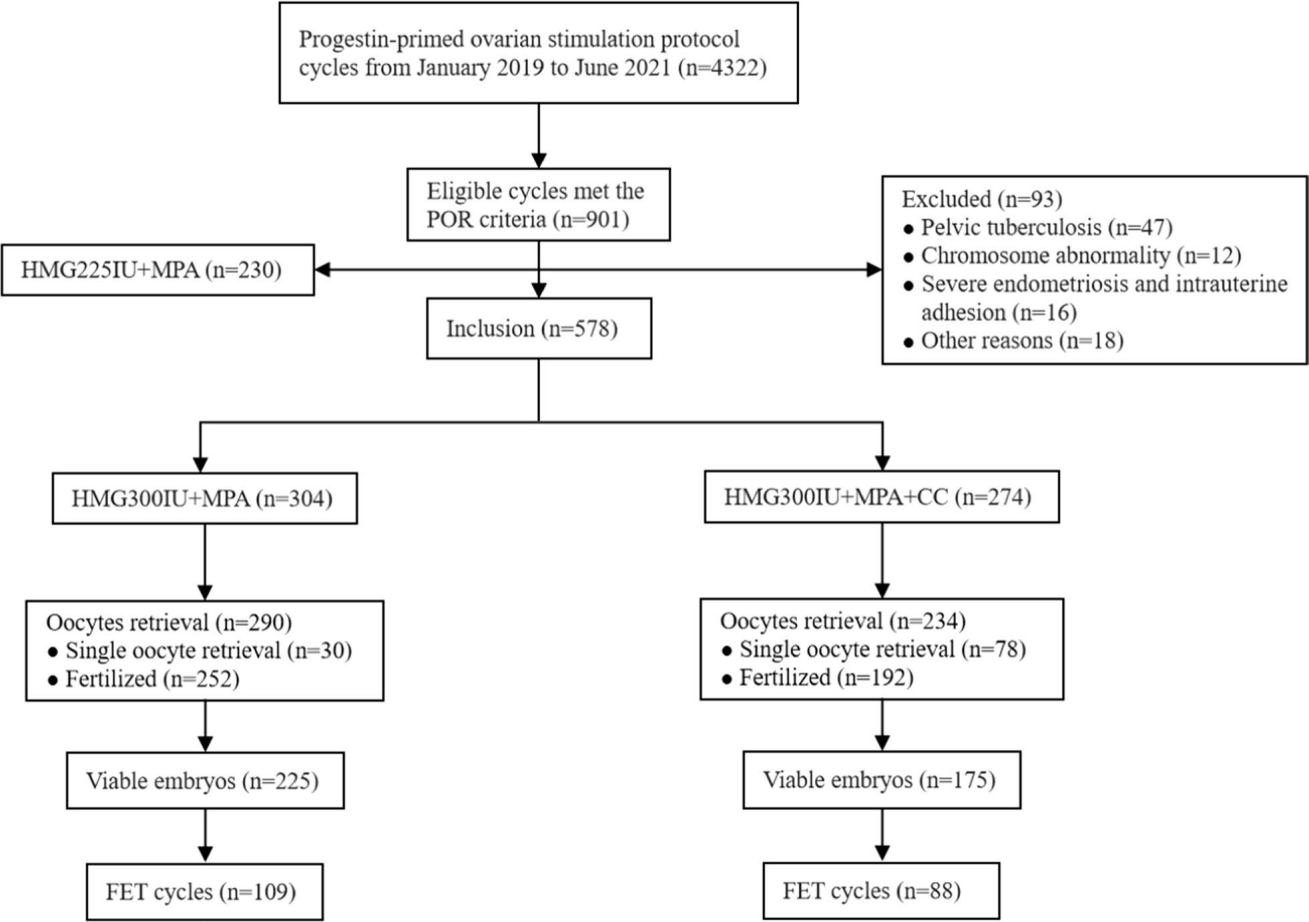
Study flow chart

Between January 2019 and June 2021 at our center, 4322 cycles received the PPOS protocol for ovarian stimulation. Of these cycles, 20.8% (901/4332) fulfilled the POSEIDON group 3 and group 4 criteria for POR, and 578 patients of these were eventually eligible for inclusion. The flowchart of the study selection is shown in Fig. 1.

The data on baseline characteristics are summarized in Table 1. In this study, 65.1% (376/578) of the POR patients met the POSEIDON group 4 criterion, and the baseline information between the two groups was not statistically significant (*P* > 0.05). The average age was 37.25 ± 5.10 years old in Group A and 36.93 ± 6.21 years old in Group B.

**Table 1:** The baseline characteristics of POR women

Characteristics	Group A (n = 304) (HMG300IU + MPA)	Group B (n = 274) (HMG300IU + MPA + CC)	*P*
Age (y)	37.25 ± 5.10	36.93 ± 6.21	0.512
POSEIDON criteria			
Group 3, n (%)	95 (31.25)	107 (39.05)	0.050
Group 4, n (%)	209 (68.75)	167 (60.95)	
BMI (kg/m^2^)	23.16 ± 2.85	23.57 ± 2.89	0.089
AMH (ng/mL)	0.46 ± 0.29	0.42 ± 0.32	0.064
AFC	2.95 ± 1.02	2.82 ± 1.03	0.085
Duration of infertility (y)	4.09 ± 3.90	4.26 ± 4.12	0.129
Primary infertility, n (%)	122 (40.13)	124 (45.26)	0.213
Cause of infertility, n (%)			
POR only	34 (11.18)	25 (9.12)	0.414
POR + tubal	174 (57.24)	154 (56.20)	0.802
POR + male	78 (25.66)	76 (27.74)	0.572
POR + other	18 (5.92)	19 (6.93)	0.619
ICSI rate, n (%)	140 (46.05)	122 (44.53)	0.713

### Ovarian stimulation and embryo performance

The clinical and cycle characteristics of the two ovarian stimulation groups are presented in Table 2. The baseline serum hormone levels were comparable between the two groups. The serum E2 level in Group B on the day of HCG was lower than that in Group A (*P* < 0.001), but the level of LH and P were significantly higher in Group B (*P* < 0.001). The total dose of HMG and duration of stimulation in Group B were significantly lower than those in Group A (*P* < 0.001). The number of oocyte for retrieval, maturation, and fertilization were significantly lower in Group B than that in Group A (*P* < 0.001). However, the oocyte retrieval rate, maturation rate, fertilization rate, and viable embryo rate showed no statistical difference in the two groups (*P* > 0.05).

**Table 2:** Clinical and cycle characteristics of ovarian stimulation in the groups

Characteristics	Group A (n = 304) (HMG300IU + MPA)	Group B (n = 274) (HMG300IU + MPA + CC)	*P*
Baseline hormones			
E2 (pg /ml)	54.47 ± 47.82	50.73 ± 43.12	0.291
FSH (mIU/l)	12.92 ± 9.37	12.98 ± 10.09	0.579
LH (mIU/ml)	5.95 ± 7.20	6.09 ± 6.82	0.672
Hormones level on the day of HCG			
E2 (pg/ml)	1376.51 ± 926.02	1093.07 ± 786.72	< 0.001
LH (mIU/ml)	2.92 ± 2.41	6.66 ± 4.34	< 0.001
P (ng/ml)	0.56 ± 0.63	0.93 ± 2.93	0.002
Total HMG doses (IU)	3399.18 ± 820.75	2998.63 ± 1051.09	< 0.001
HMG duration (days)	11.27 ± 2.56	10.21 ± 3.56	< 0.001
> 14 mm follicles on the day of HCG (n)	3.64 ± 2.06	2.75 ± 1.97	< 0.001
Retrieved oocytes (n)	3.02 ± 2.08	2.30 ± 2.22	< 0.001
Mature oocytes (n)	2.61 ± 1.93	1.98 ± 2.07	< 0.001
Fertilized oocytes (n)	1.90 ± 1.58	1.47 ± 1.68	< 0.001
Viable embryos (n)	1.48 ± 1.33	1.22 ± 1.40	0.019
High-quality embryos (n)	0.69 ± 1.00	0.52 ± 0.78	0.091
Oocyte retrieval rate n (%)	917/1107 (82.84)	631/756 (83.47)	0.722
Mature oocytes rate n (%)	793/917 (86.48)	542/631 (85.90)	0.744
Fertilization rate n (%)	578/793 (72.89)	404/542 (74.54)	0.502
Viable embryos rate n (%)	451/578 (78.03)	333/404 (82.43)	0.091

### Pregnancy outcomes after FET cycles

FET pregnancy outcomes in the two groups are presented in Table 3. Due to the poor ovarian reserve and a low oocyte retrieval rate in POR patients, which means available embryos require gradually accumulate, led the embryos transferred in the FET cycle may come from different ovulation cycles, and this condition was not eligible for inclusion in our study. Finally, two and a half years of follow-up was completed for only 197 FET cycles in the present study (109 in Group A and 88 in Group B). The mean number of transferred embryos and endometrial thickness were comparable between the two groups. Comparison of Group A and Group B revealed a similar rate of clinical pregnancy (37.61% vs. 32.95%, respectively; OR 1.230; 95% CI 0.680–2.211; adjusted OR 1.286; 95% CI 0.671–2.470), an identical rate of live birth (41.46% vs. 34.48, respectively; OR 1.353; 95% CI 0.496–3.611; adjusted OR 1.390; 95% CI 0.478–3.990). Similarly, the miscarriage rate and biochemical pregnancy rate were not significantly different between the two groups. The mean gestational age and birth weights were 37.9 ± 1.5 weeks and 3014.4 ± 357.3 g in Group A, and 37.9 ± 2.3 weeks and 2895.8 ± 393.4 g in Group B, respectively. For pregnancy outcomes in FET cycles, data were available only until June 2021. Thus, the ongoing pregnancy rate was 17.07% in Group A and 17.24% in Group B.

**Table 3:** Pregnancy outcomes after FET cycles in the groups

Outcome	Group A (HMG300IU + MPA) (n = 109)	Group B (HMG300IU + MPA + CC) (n = 88)	Group A vs Group B			
			*P*	Crude OR (95% CI)	Adjusted *P*	Adjusted OR (95% CI)
Number of transferred embryos	1.95 ± 0.45	1.86 ± 0.48	0.223	–	–	–
Endometrial thickness (mm)	9.79 ± 1.79	9.57 ± 1.82	0.395	–	–	–
Clinical pregnancy rate (%)	37.61 (41/109)	32.95 (29/88)	0.497	1.230 (0.680–2.211)	0.441	1.286 (0.671–2.470)
Live birth rate (%)	41.46 (17/41)	34.48 (10/29)	0.555	1.353 (0.496–3.611)	0.551	1.390 (0.478–3.990)
Miscarriage rate (%)	29.27 (12/41)	20.69 (6/29)	0.420	0.632 (0.210–1.942)	0.728	1.091 (0.261–4.660)
Ongoing pregnancy rate (%)	17.07 (7/41)	17.24 (5/29)	0.985	0.988 (0.280–3.487)	0.534	1.672 (0.331–8.455)
Biochemical pregnancy rate (%)	9.17 (10/109)	7.95 (7/88)	0.762	0.857 (0.311–2.346)	0.905	0.932 (0.307–2.844)
Gestational age (wk)	37.93 ± 1.50	37.87 ± 2.29	0.605	–	–	–
< 37 weeks	23.53 (4/17)	20 (2/10)	0.831	0.809 (0.120–5.496)	0.179	0.041 (0.000–4.348)
≥ 37 weeks	76.47 (13/17)	80 (8/10)	–	Ref	–	Ref
Birth weights (g)	3014.44 ± 357.26	2895.83 ± 393.39	0.545	–	–	–
Less than 2500	5.88 (1/17)	10 (1/10)	0.696	1.782 (0.102–31.976)	0.999	–
2500–4000	94.12 (16/17)	90 (9/10)	–	Ref	–	Ref

*OR* odds ratio. Adjusted for confounders, including age, AMH, BMI, AFC, E2 level on the day of HCG, retrieved oocytes, high-quality embryos, number of transferred embryos, endometrial thickness

## Discussion

The present study here aimed to evaluate the efficacy of CC supplementation in PPOS protocol for patients with POR. In our study, all included patients used the PPOS protocol, and such a choice was suitable for poor responders [17]. Our current results showed that the PPOS protocol combined with CC reduced the total dose of HMG and the duration of stimulation, which was related to the negative feedback mechanism of CC. The synergistic action by CC and HMG during the early follicular phase, made CC supplementation have significant advantages in terms of shorter duration and lower HMG consumption, and these findings were in agreement with other studies. A prospective study published in 2015 made a comparison of HMG with or without CC supplementation in ovarian stimulation for PCOS patients [18] and found that CC significantly reduced both the HMG dose and the duration of treatment. Similar results have been previously reported in another study for 320 normal ovulatory women that were divided into the HMG + MPA + CC group and the HMG + MPA group [13]. A recent meta-analysis of adjuvant treatment with CC in POR has reached the same conclusion [1]. Moreover, it must be emphasized that our study population is relevant for women with POR, who need repeating ovarian stimulation and oocyte retrieval to accumulate embryos, therefore, this advantage of CC mentioned above could relieve patients from the pain of daily injection and financial burden to some extent.

Moreover, although the AFC is similar between the two groups in baseline characteristics, the number of ovarian follicles > 14 mm on the day of HCG, oocyte retrieved, matured oocyte, and fertilized oocyte were lower in the group treated with CC. Indeed, similar results have been reported earlier, a retrospective study found that adding CC to the PPOS protocol in PCOS women showed a lower number of the retrieved and fertilized oocytes [10], they hypothesized that exogenous HMG promoted more development of medium-sized follicles, however, CC plays a role mainly by leading endogenous gonadotropins secretion in the early stage of ovarian stimulation, and this effect of CC may not be as direct as HMG, thus the use of less HMG in the CC group results in less medium-sized follicles. Their study also indicated that the number of follicles (diameters > 14 mm) in the CC group was lower while having a greater number of small-sized follicles (10–14 mm), which could also explain their hypotheses above. Ye H et al. in 2018 made a comparison of CC co-treatment with milder PPOS protocol (HMG 150 IU/d + CC 50 mg/d + MPA 10 mg/d) and PPOS (HMG 225 IU/d + MPA 10 mg/d) alone in PCOS patients and reached a result that CC led to lower oocyte yields, they interpret the results as the characteristics of milder stimulation [14]. In contrast to these studies, a prospective cohort trial included 12 POR patients that met the Bologna criteria and underwent 27 stimulation cycles of GnRH antagonist protocol and indicated that the addition of CC increased the number of oocytes retrieved and available embryos [19]. In our study, although the number of the oocyte for retrieval, maturation, and fertilization were significantly lower in Group B than that in Group A, the oocyte retrieval rate, maturation rate, fertilization rate, and viable embryos rate showed no statistical difference in the two groups.

Notably, our findings revealed that the LH level on the day of HCG was the highest in the CC supplementation group. Similarly, Jiang et al. in 2017 found that obese PCOS patients who were treated with CC had a higher LH level on HCG day [10], and the reason for this can be explained as there were some gene mutations of the LH and LH receptors in women with PCOS that made them LH-dependent during follicle development [20]. Same results have been reported in another research for PCOS patients who used the PPOS protocol with CC [14]. It is worth mentioning that previous studies have indicated that CC acts directly on the hypothalamus to increase the pulse frequency of GnRH release with normally ovulating women [21] while enhancing the amplitude of GnRH release in PCOS women [22]. It is generally known that LH plays a major role in the development and maturation of follicles [23], levels of LH that are too low or too high are associated with an increased risk of adverse clinical outcomes [24, 25]. Some studies have found that the high LH levels might be correlated with adverse ovarian stimulation outcomes such as lower rate of oocytes maturation and fertilization, impaired embryos quality [26, 27]. Shoham et al. proposed a window for LH in ovarian stimulation and believed that higher LH levels contribute to impaired folliculogenesis [28]. These may explain our results that the decreased oocytes maturation rate and fertilization rate in Group B treated with CC had a nearly three-fold higher LH level than that in Group A. On the other hand, although the LH level increased in the group added with CC, no premature LH surge occurred in the groups, and the FET pregnancy outcomes were similar between the two groups which revealed the embryos from the groups had the same developing potential.

However, in our study, the MPA was used in the PPOS protocol which can suppress pituitary LH levels. Several investigators have demonstrated the negative effects of low endogenous LH concentrations on follicle development [29–31]. Westergaard et al. showed that a low LH level (< 0.5 IU/l) on day 8 of the ovarian stimulation had a negative impact on the pregnancy outcome, and led to a significantly higher incidence of abortion in the early phase [29]. Esposito et al. also demonstrated that low levels of LH (< 3 mIU/ml) in the late phase of oocyte development may significantly cause low fertilization rates [30]. Liu et al. also reported the same phenomena for women with normal ovarian reserve applying the PPOS protocol with CC and indicated that the MPA’s application may result in stronger LH suppression, the addition of CC can alleviate this suppression without affecting the effect of MPA [13]. In other words, it may reach a balance between CC and MPA in the collective effect action to the change in LH trend during ovulation induction. Additionally, our studies did not show a detrimental effect on pregnancy outcomes that adding CC to the PPOS protocol, similar results have also occurred in women with normal ovarian reserve or PCOS who used the same stimulation regimens [13, 14]. Furthermore, a meta-analysis in 2016 by Song et al. included four randomized controlled trials and indicated that the mild stimulation protocol with CC in poor responders had the same pregnancy outcomes compared with the conventional GnRH agonist protocol [5]. Nevertheless, the findings published by Siristatidis et al. and Pilehvari et al. reported a negative effect of CC on pregnancy outcomes in POR patients. Both of the researchers compared the addition of CC to a mild stimulation protocol with conventional long-agonist or antagonist protocols [2, 32].

The limitation of this paper is the inclusion of POR patients who met only the group 3 and group 4 criteria for POSEIDON, which caused it cannot be comprehensively assessed the treatment efficacy of CC supplementation in PPOS protocol in all POR patients. Another potential limitation is that it is a retrospective study and because our study subjects were POR patients who required multiple ovulation inductions to obtain the limited number of embryos, thus the number of FET cycles was relatively small and further studies with larger sample sizes are needed to verify our findings about the different pregnancy outcomes of the two protocols.

## Conclusion

We demonstrate that the PPOS protocol combined with CC reduces the total dose of HMG and the duration of stimulation, can also achieve similar oocyte yields and clinical pregnancy rate compared with the PPOS protocol used alone in poor ovarian responders, which is more cost-saving. Our research may provide some ideas for further exploring the most suitable and patient-friendly treatment regimens for patients with poor ovarian response.

## Data Availability

All data generated or analyzed during this study are included in this published article.
